# The combined orthodontic and restorative treatment for patients with malocclusion and dentition defects: A randomized controlled trial

**DOI:** 10.1097/MD.0000000000035025

**Published:** 2023-09-01

**Authors:** Yan Shen, Xiongying Jiang, Jing Yu

**Affiliations:** a Department of Stomatology, Lianyungang Hospital of Traditional Chinese Medicine, Nanjing University of Traditional Chinese Medicine, Nanjing, China; b Department of Stomatology, Xiaoshan District Hospital of Traditional Chinese Medicine, Hangzhou, China.

**Keywords:** chewing function, dentistry, misalignment with dentition defect, oral restoration, orthodontics, restorative approach

## Abstract

**Background:**

To explore the effects of a combined orthodontic and restorative approach on chewing, swallowing, and language function in patients with malocclusion and dental defects.

**Methods:**

A total of 112 patients with malocclusion and dentition defects admitted to the Lianyungang Hospital of Traditional Chinese Medicine from June 2019 to June 2022 were prospectively selected. The patients were divided into study and control groups using a simple random number table method, with 56 patients in each group. The control group received routine restoration, whereas the study group received a combination of orthodontic and restorative treatments. The chewing function, swallowing and language function, and gingival periodontal condition before and after treatment in both groups were compared using t-test or Wilcoxon test, while treatment satisfaction were compared using chi-square test or Fisher exact test.

**Results:**

After treatment, maximum area frame bite force/max movie force in both groups increased compared to before treatment, while occlusion time, bite force distrbution balance, and standard deviation hue decreased compared to before treatment. Moreover, maximum area frame bite force/max movie force in the study group was higher than that in the control group, whereas occlusion time, bite force distrbution balance, and standard deviation hue were lower than those in the control group (*P* < .05). The swallowing and language function scores of the study group were higher than those of the control group (*P* < .05). After treatment, the bleeding index, plaque index, and probing depth of both groups decreased compared to before treatment, and the study group was lower than the control group (*P* < .05). The treatment satisfaction of the study group (94.64%) was higher than that of the control group (82.14%) (*P* < .05).

**Conclusion:**

Adopting a combined orthodontic and restorative approach to intervene in patients with malocclusion and dentition defects is beneficial for improving their periodontal condition, effectively restoring chewing, swallowing, and language functions, and achieving high patient satisfaction.

## 1. Introduction

Misalignment with dentition defects is a common type of oral disease in clinical practice, usually caused by jawbone disease, trauma, developmental disorders, periodontal disease, and other factors related to dentition loss.^[1]^ It can cause varying degrees of damage to patients vocal and chewing functions, and, to some extent, affect their daily work and life, leading to depression, anxiety, and other emotions.^[1,2]^ Taking active and effective measures to repair and treat malocclusion and dentition defects are of great significance in regulating the patient’s physical and mental state and promoting their early return to normal social work and life.^[3]^

Currently, routine restoration measures are often used in clinical practice to intervene in patients with malocclusion and dentition defects, which can achieve certain results.^[4]^ However, during the restoration period, there may be problems such as difficulty in making the restoration, mismatched appearance, etc, which make it difficult to fully meet the actual needs of patients, and the recovery status of the periodontal body is difficult to meet clinical expectations.^[4,5]^ With the deepening of clinical research, it has been found that orthodontic treatment based on conventional restoration can form a good periodontal relationship with adjacent teeth and completely preserve the alveolar bone at the top and buccal edges of the alveolar ridge, thereby improving oral chewing and occlusal function.^[6,7]^

Therefore, this study aimed to identify 112 patients with malocclusion and dentition defects in our hospital for grouping research, with the aim of clarifying the impact of a combination of orthodontic and restorative treatments on chewing, swallowing, and language function, in order to provide new insights and a reference basis for the clinical treatment of malocclusion and dentition defects.

## 2. Materials and Methods

### 2.1. General information

**Figure 1.**
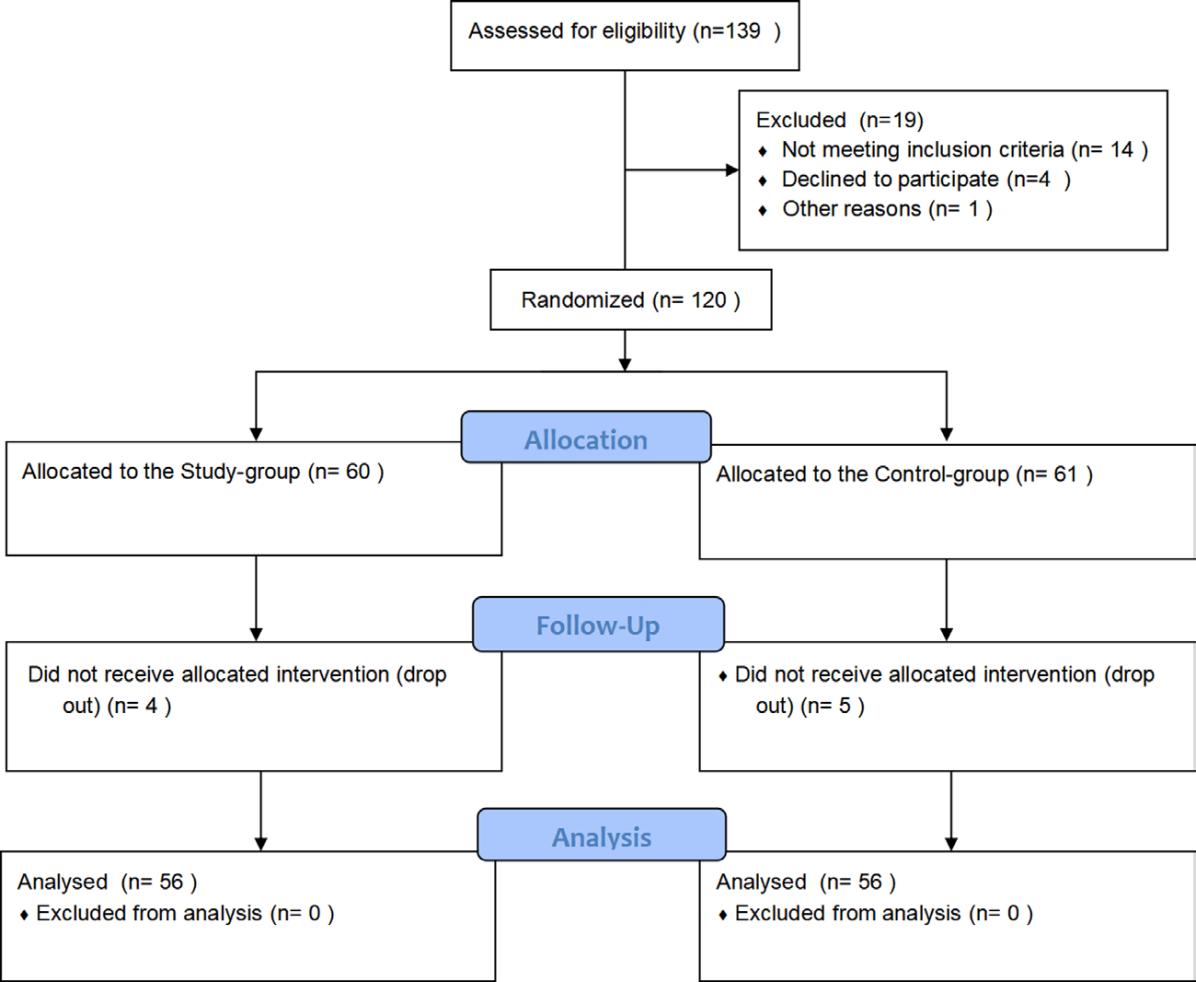
Flow chart of the participants.

A total of 112 patients with malocclusion and dentition defects admitted to the Lianyungang Hospital of Traditional Chinese Medicine from June 2019 to June 2022 were prospectively selected. They were divided into study and control groups using a simple random number table method, with 56 patients in each group (Fig. 1). There were 29 males and 27 females in the study group; Age range from 24 to 49 years, with an average age of 36.18 ± 6.09 years; Location of dentition defects: 32 cases of simple anterior teeth, 15 cases of simple posterior teeth, and 9 cases of anterior and posterior tooth defects; Classification of malocclusion: 17 cases in Class I, 28 cases in Class II, and 11 cases in Class III. There were 31 males and 25 females in the control group; Age range from 25 to 48 years, with an average age of 35.45 ± 5.70 years; Location of dentition defects: 31 cases of simple anterior teeth, 14 cases of simple posterior teeth, and 11 cases of anterior and posterior tooth defects; Classification of malocclusion: 18 cases in Class I, 29 cases in Class II, and 9 cases in Class III. The clinical data of sex, age, dentition defect location, and malocclusion classification between the 2 groups were balanced and comparable (*P* > .05).

#### 2.1.1. Inclusion criteria.

1. Age ≥ 18 years old.
2. No relevant treatment was taken within 3 months prior to inclusion in the study.
3. First time receiving treatment.

#### 2.1.2. Exclusion criteria.

1. Individuals with other oral diseases.
2. Individuals with immune dysfunction and other immune system diseases.
3. Individuals with benign and malignant tumors.
4. People with diabetes and hypertension.
5. Individuals with active bleeding.
6. People with dental caries.

All procedures performed in the study involving human participants were in accordance with the ethical standards of the institutional and/or national research committee(s) and the Helsinki Declaration (as revised in 2013). Written informed consent was obtained from the patient or legal guardian, and the medical ethics Committee of Lianyungang Traditional Chinese Medicine Hospital approved this study (2023-KY-04).

### 2.2. Methods

After admission, patients in both groups underwent routine oral examinations with X-ray system (Sirona, Bensheim, Germany), and curved tomography and cephalometric films were taken by experienced radiologists. Alveolar bone condition, area of tooth loss, inclination of tooth axis, shape of dental arch, residual teeth, pressure of tooth and groove of the patients were checked, and the periodontal and pulp disease were treated. Dental models were made according to the patient’s tooth root and alveolar bone condition, and treatment protocols were designed after full communication with the patients.

#### 2.2.1. Control group.

Underwent routine restoration and received corresponding restoration treatments based on the oral examination and the actual condition of the patient, including porcelain fused to metal crowns, crowns, and bridges.

#### 2.2.2. Study group.

Adopting a joint orthodontic and restorative plan, first implementing orthodontic treatment, followed by routine restorative interventions, with the same restorative measures as the control group. Orthodontic treatment was performed on a patient-specific basis. Straight wire arch brackets were bonded to the appropriate incisors, and then orthodontic treatment was carried out using elastic arch wires, and then the arch wires were fixed on the fixed appliance, and the dentition and bracket are pulled using rubber bands. Then we aligned and leveled the dentition, adjusted and repair the gap between teeth, corrected crossbite, and adjusted occlusal interference. After that, braces maintenance was given, and restorative therapy was implemented after the orthodontic treatment had achieved the desired efficacy. For those with sparse dentition, centralized dentures should be used to repair the dental gaps; For patients with tilted teeth, orthodontic devices should be used for correction, and denture implantation should be carried out according to the tooth gap; For patients with deep overbite, first use a guide plate appliance to lower the anterior teeth, and then perform denture restoration; For patients with tooth misalignment, the first step is to use a straight wire arch to fix and correct them. After the teeth are pulled to their normal position, the corresponding treatment should be carried out according to the tooth gap. After the repair is completed, the retainers are worn for 6 months.

### 2.3. Outcome measures

Chewing function was assessed before and after treatment in both groups, including occlusal status and chewing efficiency. The occlusal status was evaluated using a digital occlusal analysis system (T-Scan III 7.01) (Tekscan, Inc. Boston, MA).^[8]^ Appropriate occlusal films were selected according to the dental arch, the width of the maxillary central incisor, and the position of the missing teeth; guide the patient to take a sitting position, with both eyes facing up, with the orbital and ear planes parallel to the ground. The occlusal film was gently rotated into the mouth, gently touching the midline of the central incisor with the protruding point of the sensor bracket and the handle parallel to the maxillary occlusal plane–2 to 3 times. Adjust the sensitivity and detect the mandibular posture to the interdental position for occlusal separation, as well as to the left and right lateral jaw and protruded jaw. Maximum area frame bite force/max movie force (MABF/MMF), occlusion time (OT), and bite force distribution balance (BFDB) records Chewing efficiency: Take red and green chewing gum and cut it to 30 mm × 20 mm × 2 mm, stacked together as chewable material; guide the patient to take a sitting position, chew 20 times, spit it out into a transparent self-sealing bag, press the chewing material into a circular cake shape, and use a DSLR camera (Canon EOS D60, Canon Inc., Tokyo, Japan) to capture its front and back images in the same dark room, light source, and white background, combine the image into 1 image using Photoshop, and calculate the standard deviation hue (SDHue) of the standardized tone values of each pixel in the gum using ViewGum software (dHAL Software, Kifissia, Greece), with a numerical range of 0-1; The lower the SDHue, the better;^[9]^ Calculation of swallowing and language function before and after treatment in both groups, with scores ranging from 0 to 100 points; the higher the score, the better the swallowing and language functions; Periodontal status, including bleeding index (BI), plaque index (PLI), and probing depth (PD) indicators, was measured before and after treatment in both groups;^[10]^ Calculate the satisfaction level of 2 groups of treatments, create a self-made treatment satisfaction evaluation form, and evaluate the aesthetics and effectiveness of the treatment; A total of 100 points, divided into very satisfied (90–100 points), satisfied (70–89 points), dissatisfied (≤69 points), very satisfied and satisfied are included in the total satisfaction.

### 2.4. Sample size

Sample size was calculated using PASS version 21.0.3 (NCSs, LLC. Kaysville, Utah) based on the results of our preliminary experiment, 10 participants in each group, with MABF/MMF as the primary outcome. Power analysis showed a reduction rate of 10% with α = 0.05 and a 10% dropout rate within a power value of 90%, a sample size of at least 56 per group was needed. A total of 60 samples from each group were used in this study. Figure 1 showed the consolidated standards of reporting the trial flow diagram of the study participants recruitment.

### 2.5. Randomization and allocation concealment

Patients were randomly assigned to 2 groups. Random tables were generated using SPSS version 26.0 (IBM SPSS Inc., Chicago, IL). One hundred and twenty sealed envelopes were prepared by a statistician who did not participate in this study. The study was performed with neither patient nor observer awareness of the group to which each patient belonged. To ensure concealment of allocation, the numbers were kept in sealed and opaque envelopes that were opened by an anesthesiologist who was not involved in this study.

### 2.6. Statistical analysis

All data analysis was conducted using SPSS 26.0 software (IBM SPSS Inc., Chicago, IL) and GraphPad Prism 8.0 software (GraphPad Software Inc., San Diego, CA). The normality of the data was evaluated using the Shapiro–Wilk test. The data of normal distribution are expressed as mean ± standard deviation, and *t* test was used, and the data of non-normal distribution were expressed as median and interquartile interval and analyzed using the Wilcoxon test. The counting data are represented by the number of use cases using the chi-square test or Fisher exact probability method. Establishment of multivariate logistic regression model. The predictive ability of the indicators was evaluated using receiver operating characteristic curves. *P* < .05, suggesting that the difference was statistically significant.

## 3. Results

### 3.1. Masticatory function

There were no significant differences in MABF/MMF, OT, BFDB, and SDHue between the 2 groups before treatment (*P* > .05). After treatment, MABF/MMF increased compared to before treatment, while OT, BFDB, and SDHue decreased. Moreover, MABF/MMF in the study group was higher than that in the control group, whereas OT, BFDB, and SDHue were lower than those in the control group (*P* < .05) (Table 1).

**Table 1:** Comparison of chewing function between two groups.

Time	Group	n	MABF/MMF (%)	OT (s)	BFDB (%)	SDHue
Before treatment	Study group	56	90.76 ± 6.30	0.49 ± 0.15	16.05 ± 1.52	0.86 ± 0.26
	Control group	56	89.55 ± 6.46	0.48 ± 0.16	15.52 ± 1.77	0.83 ± 0.29
	*t*		1.007	0.482	1.720	0.541
	*P* value		.316	.631	.088	.589
After treatment	Study group	56	95.53 ± 3.30*	0.26 ± 0.10*	9.73 ± 1.37*	0.56 ± 0.24*
	Control group	56	91.36 ± 4.18*	0.38 ± 0.14*	11.32 ± 1.36*	0.68 ± 0.28*
	*t*		5.874	−5.218	−6.157	−2.365
	*P* value		*<*.001	*<*.001	*<*.001	.020

Compared with before treatment in this group.

BFDB = bite force distrbution balance, MABF/MMF = maximum area frame bite force/max movie force, OT = occlusion time, SDHue = standard deviation hue.

* *P* < .05.

### 3.2. Swallowing and language function

**Figure 2.**
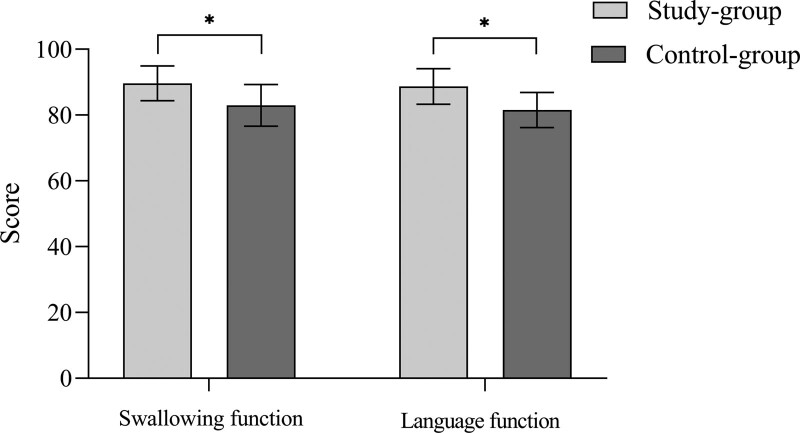
Comparison of swallowing and language function between two groups.

The swallowing and language function scores of the research group were higher than those of the control group (*P* < .05) (Fig. 2).

### 3.3. Periodontal condition

**Figure 3.**
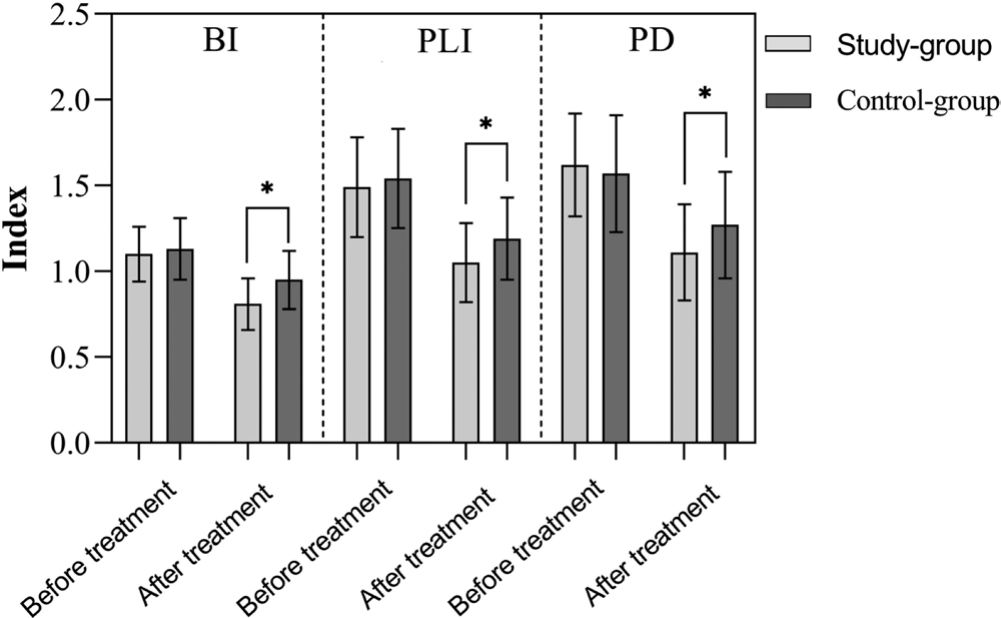
Comparison of periodontal conditions between two groups.

Before treatment, there were no significant differences in BI, PLI, and PD between the 2 groups (*P* > .05). After treatment, BI, PLI, and PD in the 2 groups decreased compared to before treatment, and the study group was lower than the control group (*P* < .05) (Fig. 3).

### 3.4. Treatment satisfaction

The treatment satisfaction of the research group (94.64%) was higher than that of the control group (82.14%) (*P* < .05) (Table 2).

**Table 2:** Comparison of treatment satisfaction between two groups.

Group	n	Very satisfied	satisfied	Dissatisfied	Overall satisfaction
Study group	56	29 (51.79)	24 (42.86)	3 (5.36)	53 (94.64)
Control group	56	24 (42.86)	22 (39.29)	10 (17.86)	46 (82.14)
*χ^2^*					4.264
*P* value					.039

## 4. Discussion

This study adopted a combined orthodontic and restorative approach to intervene in patients with malocclusion and dentition defects. The results showed that the MABF/MMF and swallowing and language function scores of the study group were higher than those of the control group, while OT, BFDB, SDHue, BI, PLI, and PD were lower than those of the control group (*P* < .05). Xie et al^[11]^ confirmed that orthodontics has a positive effect on improving oral function, pronunciation function, and facial aesthetics in patients with malocclusion. Yamamoto et al^[12]^ have shown that combining conventional restorations with orthodontic regimens can expand the upper airway, enlarge the upper airway, and prevent further deterioration of obstructive sleep apnea in patients with severe class II malocclusion; moreover, it can restore the patient’s occlusal state and, to some extent, improve their facial contour and functional deviation, gradually increasing the bite force and contact area. Xie et al^[13]^ studied the use of activated orthodontic devices for orthodontic treatment of patients with skeletal class II malocclusion and found that they can effectively improve the angle of the mandible base point at the nasal root point of the patient’s sella turcica, the angle of the lower alveolar seating point at the nasal root point of the upper alveolar seating point, the angle between the connecting line between the anterior and posterior nasal spine points and the anterior skull base plane, and the connecting line between the anterior nasal spine points and the pterygoid maxillary fissure point, which is beneficial for restoring the patient’s oral function. Avontroodt et al^[14]^ used routine restoration and orthodontic treatment measures to intervene in adolescent dental malocclusion, and the results confirmed that it cannot only improve oral function but also enhance facial aesthetics, which is beneficial for improving patient self-esteem. The study by Cheng Yanan et al^[15]^ also showed that the total effective rate of 91.55% in patients with malocclusion and dentition defects treated with combined orthodontic and restorative treatment was significantly higher than those treated with conventional restorative treatment alone. Moreover, the chewing function, oral hygiene, and dental condition of the patients in the study group were better than those in the control group, and the research results and conclusions of the above studies are consistent with this study, confirming that orthodontic and restorative methods have high application value in the treatment of malocclusion and dentition defects, and can effectively improve the periodontal condition, chewing function, swallowing function, and language function.^[16]^ Additionally, other variables could have a significant influence. In fact tooth brushing movement^[17]^ and probiotics have been demonstrated to have an influence on plaque and other periodontal parameters in pediatric,^[18]^ periodontal,^[19]^ and surgical^[20]^ patients. These techniques and compounds should be evaluated in combination with orthodontic treatment in future clinical trials. Misalignment with dentition defects can cause teeth to tilt and shift, resulting in abnormal jawbone morphology and size. Patients often experience varying degrees of facial beauty damage, pronunciation dysfunction, periodontal tissue changes, decreased chewing function, and may even cause temporomandibular joint lesions, posing a huge threat to the quality of life and physical and mental health of patients.^[16,21]^ Although simple restorative treatment can improve the patient’s oral occlusal status to a certain extent, owing to the presence of dental deformities, it is difficult to make suitable restorations. Even if they can be made, patients are prone to postoperative problems such as asymmetry.^[22,23]^ Orthodontic treatment is an important auxiliary intervention measure for restorative treatment, which can solve problems such as tooth alignment and improve the shape of the dental arch and dentition. The combination of the 2 can effectively restore the normal physiological function of teeth and jaws. At the same time, malocclusion of teeth and other deformities can cause changes in mandibular stress, leading to natural tooth inclination towards the gap, affecting the patient’s chewing function to varying degrees, resulting in incomplete occlusion, food impaction, and further exacerbating the condition; Implementing orthodontic treatment before restoration can adjust the position of the teeth and jaw, improve the occlusal relationship to a certain extent, and eliminate occlusal trauma; To ensure the effective restoration function of the implant and achieve the goal of improving the oral environment and chewing function.^[24,25]^ Wu Yangxuan et al^[26]^ also showed that combining orthodontic repair and implant correction for patients with dental defects and malocclusion can not only improve dental crowding and provide a good foundation for implant treatment, but also eliminate interference, improve implant stability, and avoid food impaction and abnormal bite force.

This study also conducted a survey and statistical analysis on the subjective satisfaction of patients, and the results showed that the treatment satisfaction of the study group (94.64%) was higher than that of the control group (82.14%) (*P* < .05). This indicates that patients with malocclusion and dentition defects are highly satisfied with the combined orthodontic and restorative treatment plan, mainly because this combined treatment can effectively improve the patient’s oral function and environment. It also has a certain positive significance in improving facial aesthetics; therefore, patient satisfaction is higher.

However, this study also has certain limitations, such as the relatively small sample size and the inability to observe and analyze the long-term periodontal health of the 2 groups due to the impact of the follow-up time. Furthermore, only several indicators were analyzed in this study, indicators such as debris index, calculus index, and the quality of life of the patients underwent orthodontic and restorative treatment were not analyzed. We plan to continue supplementing and improving the next stage of research.

## 5. Conclusion

Adopting a combined orthodontic and restorative approach to intervene in patients with malocclusion and dentition defects is beneficial for improving their periodontal condition, effectively restoring chewing, swallowing, and language functions, and achieving high patient satisfaction.

## Author contributions

**Conceptualization:** Yan Shen.

**Data curation:** Yan Shen, Xiongying Jiang.

**Formal analysis:** Xiongying Jiang.

**Writing – original draft:** Yan Shen.

**Writing – review & editing:** Jing Yu.
